# Ultimate Spatial Resolution Realisation in Optical Frequency Domain Reflectometry with Equal Frequency Resampling

**DOI:** 10.3390/s21144632

**Published:** 2021-07-06

**Authors:** Zhen Guo, Gaoce Han, Jize Yan, David Greenwood, James Marco, Yifei Yu

**Affiliations:** 1 Warwick Manufacturing Group (WMG), University of Warwick, Coventry CV4 7AL, UK; Zhen.Guo@warwick.ac.uk (Z.G.); Gaoce.Han@warwick.ac.uk (G.H.); D.Greenwood@warwick.ac.uk (D.G.); James.Marco@warwick.ac.uk (J.M.); 2 Electronics and Computer Science (ECS), University of Southampton, Southampton SO17 1BJ, UK; J.Yan@southampton.ac.uk

**Keywords:** distributed optical fibre sensor, optical frequency domain reflectometry (OFDR), spatial resolution, nonlinear frequency sweeping

## Abstract

A method based on equal frequency resampling is proposed to suppress laser nonlinear frequency sweeping for the ultimate spatial resolution in optical frequency domain reflectometry. Estimation inaccuracy of the sweeping frequency distribution caused by the finite sampling rate in the auxiliary interferometer can be efficiently compensated by the equal frequency resampling method. With the sweeping range of 130 nm, a 12.1 µm spatial resolution is experimentally obtained. In addition, the sampling limitation of the auxiliary interferometer-based correction is discussed. With a 200 m optical path delay in the auxiliary interferometer, a 21.3 µm spatial resolution is realised at the 191 m fibre end. By employing the proposed resampling and a drawing tower FBG array to enhance the Rayleigh backscattering, a distributed temperature sensing over a 105 m fibre with a sensing resolution of 1 cm is achieved. The measured temperature uncertainty is limited to ±0.15 °C.

## 1. Introduction

High-resolution and long-distance sensing are two essential and trade-off factors in distributed optical fibre sensors (DOFSs) [1,2,3,4,5]. Large-scale DOFSs, such as structural health-monitoring and pipeline/cable-detecting DOFSs, mainly focus on improving the signal-to-noise ratio of the backscattering light over the long-distance transmission [6,7,8]. On the other hand, high-resolution DOFSs have also attracted attention in biomedical image and optical components metrology [9,10,11]. Recently, the battery management system (BMS) is becoming one of the promising applications for high-resolution DOFSs [12]. It is essential for the BMS to accurately estimate and predict the real-time states for battery management. As stated in [13], sensing with high accuracy as well as high temporal and spatial resolution is essential to ensuring the safe and reliable operation of batteries. In recent years, point/quasi-distributed optical fibre sensing methods based on fibre Bragg grating (FBG) have been investigated and discussed. By incorporating FBG sensors into the commercial battery cell, heat capacity contribution is readily assessed for full parameterisation of the thermal modal [13]. A hybrid sensing network of FBG and Fabry–Perot cavities is embedded in a Li-ion pouch cell for strain and temperature measurement and discrimination [14]. Recently, a Rayleigh scattering based DOFS has been delivered to provide a measurement of both the in-plane temperature difference across the cell surface and the movement of the hottest region of the cell during the operation of an A5-size LiB pouch cell in real time [15]. It is believed that, with the increasing demand for estimating the state of charge/health/power of larger format battery cells and for a battery pack that includes hundreds or even thousands of battery cells, actual DOFSs with high resolution will become more competitive.

Optical frequency domain reflectometry (OFDR) is one of the Rayleigh backscattering-based DOFSs. Compared to conventional optical time domain reflectometry (OTDR), OFDR has excellent spatial resolution and dynamic range [16]. Generally, Rayleigh-based coherent OFDR employs a laser source whose frequency is tuned linearly in time. However, the nonlinear laser frequency tuning and intrinsic phase noise limit the practical resolution of the coherent OFDR [17]. An auxiliary interferometer (AI) is an effective way to estimate and eliminate the tuning nonlinearity [18]. A customised circuit is proposed to generate an external clock with higher beating frequency based on a zero-crossing detection [19]. Hilbert transformation-based correction provides another straightforward solution to suppressing the nonlinear tuning by obtaining the instantaneous sweep frequency [20]. To further improve phase stability in the optical coherence tomography (OCT), each frequency sweep of the laser is calibrated by using the Hilbert transformation [21]. An alternative complex finite impulse response (FIR) filter is employed to extract the phase of the auxiliary interferometer instead of the Hilbert transform, which is beneficial to the digital hardware efficiency [22]. Cubic spline interpolation is applied to resample the beat signal from the main interferometer to improve performance. A 0.3 mm spatial resolution over a 300 m single-mode fibre is demonstrated. The measured temperature resolution is 0.7 °C with a 7 cm sensing resolution [23]. The trade-off between effective sensing segment size and minimal measurable temperature variation is optimised in the cryogenic environment. With a sensing resolution of 8 cm, a minimal measurable temperature reaches 0.21 °C [24]. Calibration of the time-scale factor is proposed for the nonlinear frequency sweeping suppression. A spatial resolution of 0.17 mm over a 155 m fibre is obtained [25]. A high-birefringence polarisation-maintaining fibre is used for solving the temperature and strain discrimination. Temperature measurement error is limited to 0.8 °C with a sensing resolution of 6.5 mm over a 170 m fibre [26]. By eliminating the impact of birefringence non-uniformity in the polarisation-maintaining fibre, the temperature measurement uncertainty is improved to ±0.8 °C [27]. However, the realisation of theoretical spatial resolution of OFDR is still challenging due to the inaccurate estimation of frequency sweeping derived from the auxiliary interferometer-based correction, which will further hamper the improvement of sensing accuracy.

In this paper, the nonlinearity of frequency sweeping and the uncertainty of the frequency interval are investigated in the following section. An equal frequency resampling method is then proposed to provide a more accurate equal frequency interval for the theoretical spatial resolution realisation. In this study, a spatial resolution of 12.1 µm is achieved under a 130 nm frequency tuning range. The Nyquist limit of the fibre length in the auxiliary interferometer is discussed and overcome by employing the Hilbert transform. Using the Rayleigh backscattering spectrum and the cross-correlation, a distributed temperature sensing with a hundred-meter measurement range and a cm-level sensing resolution is implemented. A ±0.15 °C temperature uncertainty obtained in the distributed temperature sensing experimentally demonstrates the efficient suppression of the nonlinear frequency sweeping noise.

## 2. Principle of Equal Frequency Resampling

**Figure 1:**
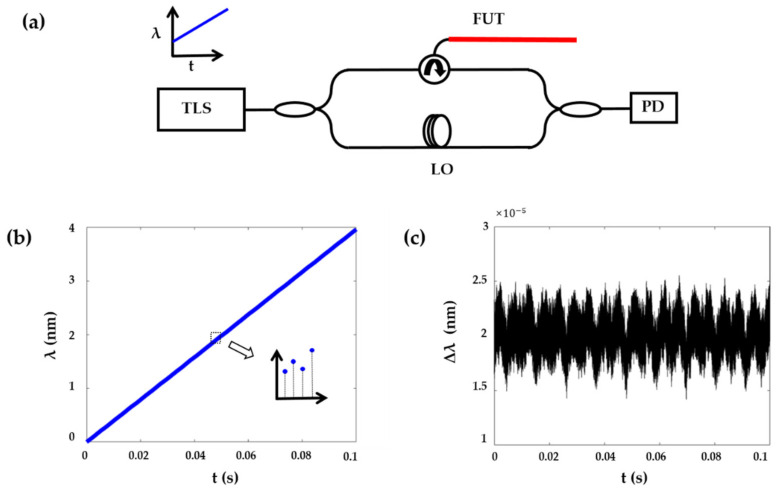
(**a**) Schematic diagram of OFDR. (**b**) Distribution of frequency sweeping at a speed of 40 nm/s. Inset: nonlinear sweeping. (**c**) Deviation of the sweeping wavelength.

The basic configuration of OFDR consists of a tuneable laser source (TLS) and an optical interferometer (Figure 1a). The local oscillator (LO) is a fixed reference arm, while the fibre under test (FUT) is in the sensing arm. The interference between reference and sensing arms is detected by a photodetector (PD). Assuming the frequency tuning speed of the laser source is γ, the interference between the position *z* along the FUT and LO can be expressed as

(1) $$I\left(t\right)=E_{0}^{2}\left\{1+\frac{2R_{LO}R_{RBS}}{R_{LO}^{2}+R_{RBS}^{2}}\cos\left[2\pi \left(\nu_{0}\tau_{z}+\gamma \tau_{z}t-1/2\gamma \tau_{z}^{2}+\phi_{n}\left(t,\tau_{z}\right)\right)\right]\right\}\;\;\;\;\;\;\;\;\;$$

where $\nu_{0}$ is the initial frequency$;\;\phi_{n}\left(t\right)$ is the phase noise; $R_{LO}$ and $R_{RBS}$ are the intensity coefficients of the LO and the Rayleigh backscattering light, respectively; and $\tau_{z}$ is the optical time delay between the position *z* and the reference arm. Thus, a position of *z* along the FUT would correspond to the beat frequency of $\gamma \tau_{z}$. Due to the limited frequency tuning range, the theoretical spatial resolution limitation of OFDR is

(2) $$\;\Delta z=c/2n\nu_{scan}$$

where c is the light speed, n is the index of fibre and $\nu_{scan}$ is the frequency tuning range.

Nonlinear frequency sweeping of the laser source is the prominent noise to deteriorate the spatial resolution of OFDR. The distribution of frequency sweeping can be measured by an interferometer and the Hilbert transform [20]. The experimental distribution of the frequency sweeping is shown in Figure 1b at a claimed tuning speed of 40 nm/s. According to Equation (2), a 4 nm tuning range corresponds to a 0.21 mm spatial resolution. The inset in Figure 1b presents the sweeping nonlinearity, which would cause frequency uncertainty and broaden the spatial resolution. To quantitatively illustrate the nonlinearity of the frequency sweeping, Figure 1c represents the wavelength deviation of the tuneable laser source: $\Delta \lambda =\left(\lambda \left(t_{2}\right)-\lambda \left(t_{1}\right)\right)\times f_{s}\cdot nL/c$, where $f_{s}$ is the sampling rate and L is the optical path delay of the interferometer (200 m). Random deviation of the sweeping wavelength is about $\pm 0.5\times 10^{-5}\;\mathrm{nm}$ at the 40 nm/s tuning speed.

**Figure 2:**
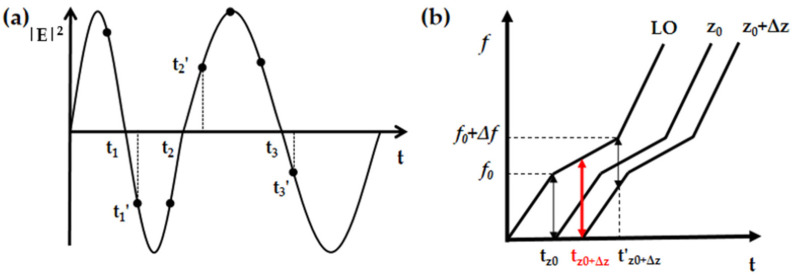
(**a**) Zero-crossing resampling with auxiliary interference fringes. $t_{1}$,$t_{2}$,$t_{3}$: zero-crossing points; $t_{1}^{\prime }$,$t_{2}^{\prime }$,$t_{3}^{\prime }$: experimental zero-crossing points; (**b**) inaccurate estimation of zero-crossing resampling.

An auxiliary interferometer is usually employed to suppress the nonlinear frequency sweeping [18]. The schematic diagram is shown in Figure 2a. Interference fringes in the fixed auxiliary interferometer usually have random frequency distribution caused by the nonlinear frequency sweeping. Zero-crossing points, shown as $t_{1}$, $t_{2}$ and $t_{3}$, are the points where the sign of the interference fringe changes from positive to negative or from negative to positive. These zero-crossing points are assumed as symbols for the same frequency interval so that they are used for resampling of the OFDR signals to compensate the sweeping nonlinearity. In an actual system, the beating frequency of the auxiliary interferometer has to meet the Nyquist theorem, which usually requires four times the fibre length compared to the length of FUT. Moreover, the finite sampling rate also restrains the estimation accuracy. As shown in Figure 2a, $t_{1}^{\prime }$, $t_{2}^{\prime }$ and $t_{3}^{\prime }$ are usually regarded as the zero-crossing points instead.

The frequency interval in the zero-crossing resampling is an estimation of the frequency distribution by the zero-crossing points. It is different from the physical process of OFDR, in which the interference is the sum of all the individual backscattering points along the FUT and the LO light. Assuming that there are two backscattering points, $z_{0}$ and $z_{0}+\Delta z$ with the nonlinear tuneable laser source (Figure 2b), actual interference will happen at $t_{z0}$ and $t_{z0+\Delta z}$, respectively. However, in zero-crossing resampling, $f_{0}+\Delta f$ is selected to resample the interference at $t_{z0+\Delta z}^{\prime }$, where Δf is the frequency interval determined by the adjacent zero-crossing points in the AI interference fringes.

Thus, the frequency interval is essential to realising the theoretical spatial resolution. Here, we propose an equal frequency resampling to compensate for the inaccurate estimation of the frequency distribution. Instead of dealing with the adjacent zero-crossing points for resampling, we calculate and optimise the instantaneous optical frequency of the TLS based on the frequency sweep distribution. Equal frequency resampling consists of two steps. The first is to calculate the instantaneous optical frequency distribution of the tuneable laser by using the auxiliary interferometer. Subsequently, the frequency interval is optimised by the overall frequency distribution.

The instantaneous optical frequency distribution can be derived by

(3) $$\nu \left(t\right)=arctan\left(\frac{H\left\{I\left(t\right)\right\}}{I\left(t\right)}\right)/\left(2\pi \tau \right)$$

where H{} is the Hilbert transform and $I\left(t\right)$ is the signal from the auxiliary interferometer. In the auxiliary interferometer-based zero-crossing resampling, the phase interval of the adjacent zero-crossing points is π, and the corresponding frequency interval is

(4) $$\Delta \nu \left(t\right)=\int_{t}^{t+\Delta t}\gamma \left(t\right)dt=\int_{t}^{t+\Delta t}\frac{1}{2\pi \tau }\frac{d\phi \left(t\right)}{dt}dt=\frac{1}{2\tau }$$

Thus, with the instantaneous frequency distribution, various frequency intervals (as π/2, π/4, π/8, …) can be obtained to overcome the Nyquist theorem limitation of the optical fibre length in the auxiliary interferometer.

Taking nonlinear sweeping into consideration, the phase part of Equation (1) can be Taylor expanded:

(5) $$\phi \left(t\right)-\phi \left(t-\tau \right)=2\pi \tau \nu \left(t\right)-2\pi \overset{\infty }{\underset{i=2}{\sum }}\frac{{\left(-\tau \right)}^{i}}{i!}\frac{d^{i-1}\nu \left(t\right)}{dt^{i-1}}$$

Usually, the second or higher-order terms $\overset{\infty }{\underset{i=2}{\sum }}\frac{{\left(-\tau \right)}^{i}}{i!}\frac{d^{i-1}\nu \left(t\right)}{dt^{i-1}}$ are neglected. However, for the ultimate spatial resolution, this approximation will induce the inaccurate estimation shown in Figure 2b. Thus, the higher term of $\pi \tau^{2}\frac{d\nu \left(t\right)}{dt}$ is utilised in the proposed equal frequency resampling to calculate the instantaneous frequency distribution. The instantaneous value of $\nu \left(t\right)$ will be modified according to

(6) $$\nu \left(t\right)=\nu_{t-t_{0}}+\overset{t}{\underset{t-t_{0}}{\int }}\gamma \left(t\right)dt=\nu_{t-t_{0}}+\overset{t}{\underset{t-t_{0}}{\int }}\frac{1}{2\pi \tau }\frac{d\phi \left(t\right)}{dt}dt$$

Frequency distribution is derived and modified by combining Equations (5) and (6). Similar to the interference between individual backscattering points along the FUT and the fixed LO arm, the frequency interval is modified by the distribution of the frequency sweeping. The equal frequency interval can therefore be guaranteed, and the theoretical-level spatial resolution can be realised.

## 3. Experimental Setup and Results

### 3.1. Theoretical Spatial Resolution Realisation by Equal Frequency Resampling

**Figure 3:**
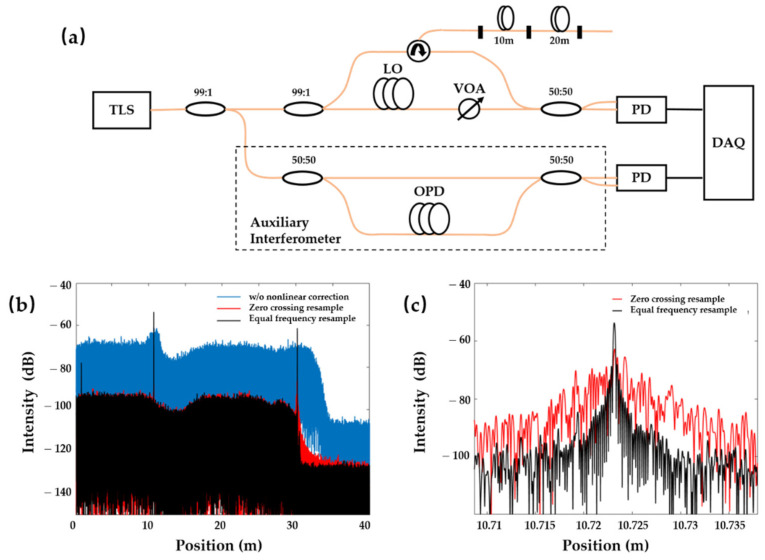
(**a**) Experimental setup; (**b**,**c**) resolution with different nonlinear sweeping corrections.

The experimental setup of the equal frequency resampling is shown in Figure 3a. A Santec-TSL-550 is used as the tuneable laser source with an output power of 9.2 dBm. Tuning speed is set at 40 nm/s. A 99/1 optical coupler splits the frequency tuning light into the main (99%) and auxiliary (1%) interferometers, respectively. The auxiliary interferometer is utilised to estimate and compensate for the nonlinear frequency sweeping of the laser source. Its optical path delay is 200 m. The main interferometer contains another 99/1 optical coupler, by which 99% of light launches through the circulator into the FUT. The Rayleigh backscattering light from the FUT will interfere with the LO light and be detected by a balanced photodetector. The variable optical attenuator (VOA) is used to adjust the interference between them. Two 99/1 couplers are implemented to improve the interference visibility. A PicoScope 6404D with 8 bits ADC resolution and 2 GS buffer size is utilised for data acquisition. Its sample rate is 78.13 MHz.

The length of the FUT is 30 m, composed of two segments (10–20 m). Fresnel reflection at the interface of two polished angle connectors is considered as an index change with ultimate spatial resolution. Thus, there should be three peaks in the OFDR spectrum. While the sweeping frequency range is 4 nm, the corresponding theoretical spatial resolution is 0.21 mm. Figure 3b shows the experimental results with/without nonlinear sweeping correction. The resolution without nonlinear sweeping correction at 10 m is approximately 1.5 m. Prior reflection at 1 m is even unable to be distinguished due to the nonlinear noise. Figure 3c presents the comparison between two correction methods. With the zero-crossing resampling, the spatial resolution at 10 m is reduced to 4.2 mm. Fluctuated chaotic peak distribution indicates the mixing of unwanted frequency from the nonlinear frequency sweep. With the equal frequency resampling, the spatial resolution at 10 m is further reduced to 0.4 mm, which is narrower than one-tenth of its size, by the zero-crossing resampling. This proves that equal frequency resampling can efficiently suppress the nonlinear noise and improve the spatial resolution to the theoretical level.

**Figure 4:**
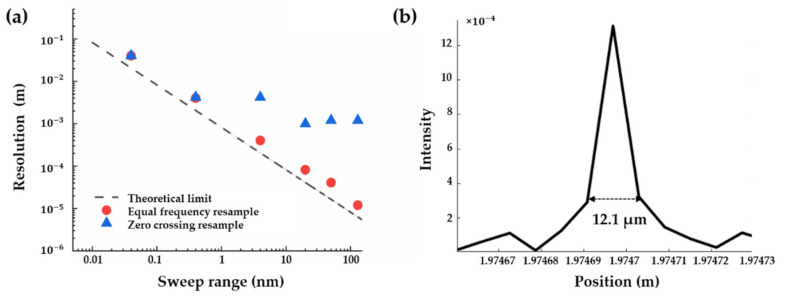
(**a**) Theoretical and experimental spatial resolution with various frequency sweeping ranges; (**b**) 12.1 µm spatial resolution with a 130 nm sweeping range.

The theoretical spatial resolution of OFDR is determined by the range of frequency sweeping, which is related to the sweeping speed of the laser source and data acquisition. Equal frequency resampling is further investigated and verified with various frequency sweeping ranges, as shown in Figure 4. Sweeping ranges of 0.04 nm, 0.4 nm and 4 nm are tested under a 40 nm/s tuning speed, while 20 nm, 50 nm and 130 nm sweeping ranges are tested under a 100 nm/s tuning speed for calculation consumption. This shows that there is little difference between zero-crossing and equal frequency resampling, while the expected spatial resolution is higher than 1 mm, which means that the frequency uncertainty caused by nonlinear sweeping and phase noise is less than the 1 mm frequency resolution. With further reduced spatial resolution, the zero-crossing resampling is unable to suppress the nonlinear frequency sweeping, while the equal frequency resampling could improve the spatial resolution from 1 mm to 12.1 µm with a theoretical spatial resolution of 6.3 µm (with a corresponding 130 nm sweeping range). To the best of our knowledge, this is the first realisation of 10-µm-level spatial resolution in OFDR interrogation. Besides the theoretical limit prediction, the distribution of position peak is clear and uniform (Figure 4b). Thus, it is demonstrated that a further improved spatial resolution could be obtained by a larger frequency sweeping range and/or faster data processing.

**Figure 5:**
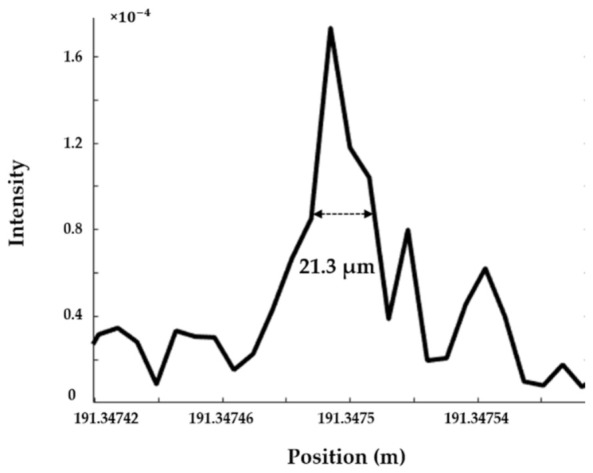
The 21.3 µm spatial resolution at 191 m with 200 m optical path delay in the auxiliary interferometer.

In the zero-crossing resampling correction, the resampling rate in the fixed auxiliary interferometer should fulfil the Nyquist theorem, requiring four times the length of the optical path delay (OPD) in the auxiliary interferometer to the FUT, which hampers long-distance sensing. With the Hilbert transform in equal frequency resampling, this Nyquist limitation can be overcome by decreasing the frequency interval. Another experiment is carried out with a 200 m FUT in the main interferometer and a 200 m OPD in the auxiliary interferometer. The sweeping wavelength range of the laser is 130 nm. With a 200 m FUT in the zero-crossing resampling, the expected length of OPD should be at least 800 m. The experimental result proves that the zero-crossing resampling is unable to detect any information around a 200 m fibre end, while the spatial resolution at a 191 m fibre end remains 21.3 µm with the equal frequency resampling (Figure 5). It is noted that the frequency interval is set at π/4 to detect the index change at the end of the 191 m fibre.

### 3.2. Distributed Temperature Sensing with High Sensing Resolution

Due to the non-uniform distribution of the index refraction along with the fibre core, FUT can be regarded as a sequence of random Bragg gratings. Thus, the applied temperature or strain will cause the shift in the local Rayleigh backscattering spectrum (RBS) [28]. OFDR can measure this RBS shift by tuning the incident wavelength for the distributed sensing. There are three steps in the OFDR sensing demodulation. First, the measured signal is transformed from the optical frequency domain to the spatial domain using the Fourier transform. As RBS cannot measure the absolute temperature/strain, it requires two optical frequency domain signals under different temperatures and/or strains, noted as the reference signal and the measurement signal. Second, a designed window is used to filter the spatial domain signal at a specific position. The length of the window is defined as the sensing resolution of the OFDR. The local RBS of each fibre segment is obtained by performing an inverse Fourier transform of the extracted spatial domain signal. Finally, the spectrum shift between the reference and measurement RBSs can be calculated by cross-correlation. This frequency shift reflects the variation in sensing parameters, such as temperature and strain. The proposed equal frequency resampling is utilised to suppress the nonlinear frequency sweeping before the first step of the Fourier transform. High spatial resolution not only provides accurate position information but also enables more data acquisition under the finite sensing resolution, which improves the signal-to-noise ratio in the fixed-length window filtering.

**Figure 6:**
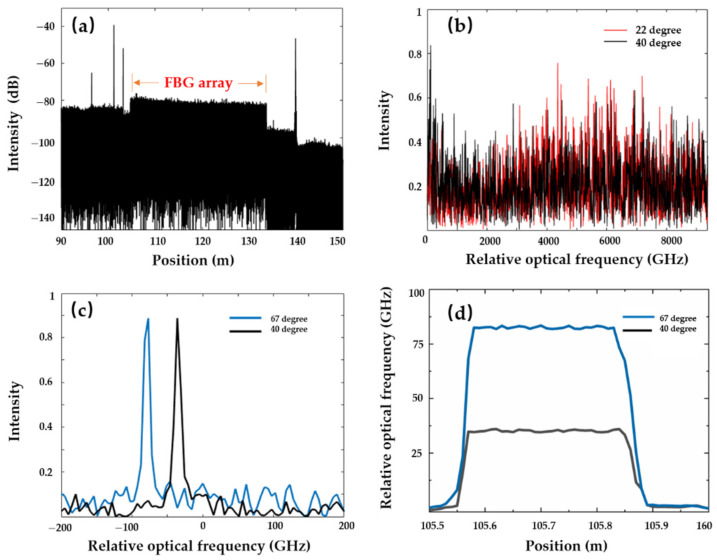
(**a**) Spatial domain distribution with a 30 m FBG array at 100 m fibre end; (**b**) RBS response at 105.77 m; (**c**) RBS shift compared to the reference of ambient 22 °C; (**d**) distributed temperature measurement with 0.01 m sensing resolution.

Non-uniform distribution of the index refraction in the fibre induces the Rayleigh backscattering. Compared to the conventional optical fibre, the drawing tower FBG array is employed to enhance the Rayleigh backscattering. A single FBG length is 10 mm, and the grating separation (from middle to middle) is also 10 mm. The central wavelength is 1550 nm, and the reflectivity of each FBG is limited to below 0.1%. The total length of the FBG array is 30 m, lying at the 100 m fibre end. The head section of a 30 cm FBG array is placed in the water bath. The sweeping wavelength range is 75 nm (1555–1630 nm), corresponding to the theoretical spatial resolution of 10.96 µm. The tuning speed is 100 nm/s. The OPD in the auxiliary interferometer is 200 m. The frequency interval in the equal frequency resampling is π/4. It is noted that the zero-crossing resampling is still unable to detect any useful information beyond a 100 m length of FUT. The initial ambient temperature is 22 °C. Interference signals from the main interferometer are compensated for and analysed by the proposed equal frequency resampling and the Fourier transform (Figure 6a). Peaks around 100 m and 140 m are the corresponding optical connectors. 

Interference at ambient 22 °C is recorded as the reference signal, while interference at 40 °C is recorded as the measurement signal. The length of the filtering window for the RBS is 0.01 m, containing 912 points of spatial resolution. Increasing the wavelength sweeping range helps to reduce the spatial resolution to tens of µm. Meanwhile, this influences the optical frequency measurement resolution:

(7) $$\delta \nu =\nu_{scan}/M$$

where M is the number of points in the filtering window. Due to the 75 nm wavelength sweeping range and 912 points in the 0.01 m window length, the minimum detected frequency shift is 10.28 GHz. In order to improve the sensing accuracy, padding zeros to the filtered spatial domain signals can improve the optical frequency measurement resolution [5]. Both reference and measurement RBSs at the 105.77 m position are shown in Figure 6b. The RBS varies with the increasing temperature. By calculating the cross-correlation of 22 °C’s and 40 °C’s RBSs, the RBS shift is 35.93 GHz. With an increased temperature of 67 °C, the corresponding RBS shift is 82.21 GHz (Figure 6c). The measured temperature distribution of the 30 cm fibre in the water bath is shown in Figure 6d. The average RBS shift in the fibre segment at 40 °C is 35.15 GHz; the deviation is limited to ±0.73 GHz. At the temperature of 67 °C, the average RBS shift and deviation are 82.54 GHz and ±0.81 GHz, respectively.

**Figure 7:**
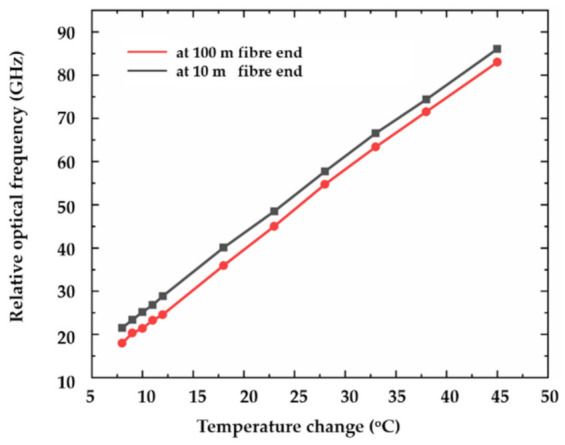
Relationship between temperature changes and the measured relative optical frequency.

The thermal sensitivity coefficient of the utilised FBG array is further investigated by gradually changing the applied temperature. The average value of the optical frequency shift in the fibre section in the water is used to avoid sensing uncertainty and deviation. Interference at the ambient temperature of 22 °C is regarded as the reference signal. The relationship between the temperature change and optical frequency shift is shown in Figure 7. The slope of the curve represents the thermal sensitivity coefficient, indicating the linearity between the temperature change and the RBS shift. The average slope of the fibre segment at 10 m fibre end is 1.76 GHz/°C, while the average slope at 100 m fibre end is 1.78 GHz/°C. R-square, the coefficient of determination, is introduced to represent the linearity of the thermal sensitivity coefficient. The value of R-square is 0.9995 at the 10 m fibre end and 0.9993 at the 100 m fibre end.

### 3.3. Uncertainty of Distributed Sensing in OFDR

For absolute temperature sensing, two measurements of Rayleigh backscattering spectrums need to be calculated and compared for the frequency shift. Spatial domain signals after the Fourier transform are designed to correspond to the same physical fibre segment. The nonlinear wavelength tuning, intrinsic laser phase noise and low Rayleigh backscattering signal-to-noise ratio induce the uncertainty of spatial domain signals. Moreover, the minimum detectable temperature variation is also limited by the optical frequency measurement resolution (Equation (7)) and the thermal sensitivity coefficient.

**Figure 8:**
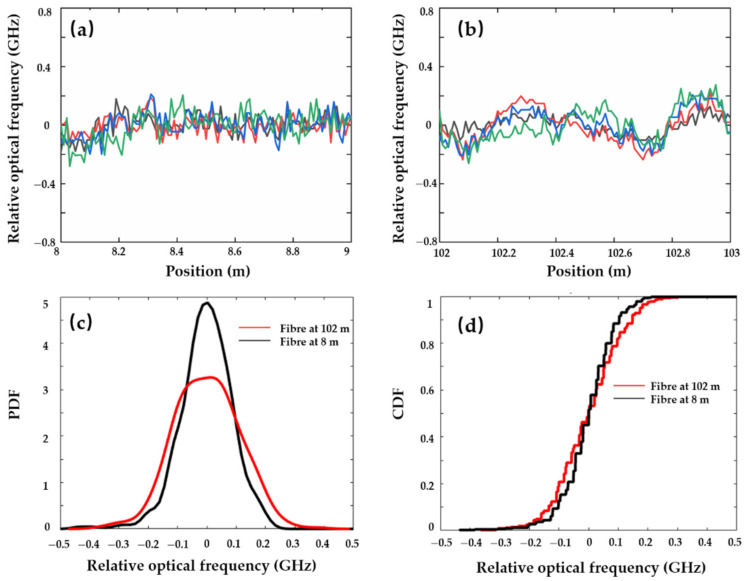
(**a**) Four RBS shift measurements at the position of 8 m; (**b**) four RBS shift measurements at the position of 102 m; (**c**) probability density function (PDF) of RBS shift at various positions; (**d**) cumulative distribution function (CDF) of RBS shift at various positions.

In order to quantify the uncertainty of distributed sensing, the same segment of fibre is tested multiple times at the same temperature. Compared to the water, the temperature distribution is more uniform and stable in the ambient air space. Thus, two fibre segments at 8 m and 102 m are simultaneously tested under the 75 nm wavelength tuning range and 200 m OPD. Four measurements of temperature distribution profile with a 0.01 m sensing resolution are shown in Figure 8a,b. The probability density function (PDF) and cumulative distribution function (CDF) are employed to represent the sensing uncertainty. The deviation of frequency shift at 102 m is worse than that of the front part at 8 m due to the phase noise and an increased nonlinear sweeping noise (as shown in Figure 5). Uncertainty of measurement is usually defined as the half-width of the optical frequency variation from 0.01 to 0.99 in the CDF curve. In Figure 8d, the measured optical frequency uncertainty is 468 MHz and 554 MHz at 8 m and 102 m, respectively. Regarding the thermal sensitivity coefficient of 1.77 GHz/°C, the corresponding temperature measurement uncertainty is 0.13 °C at 8 m and 0.15 °C at 102 m. 

In terms of the temperature uncertainty in the water, the deviation is as high as ±0.81 GHz at 67 °C. There are mainly two reasons for this: the non-uniformity of temperature distribution in the water bath and the strain/bend cross-sensitivity. The temperature is controlled by the central thermostats in the water bath, rendering it difficult to form an evenly distributed temperature environment while maintaining a high temperature. Moreover, the process of heating will induce the unexpected water flow to bend the optical fibre. 

### 3.4. Further Sub-µm-Level Spatial Resolution

Accurate estimation of frequency sweeping is the key to realising ultimate spatial resolution with the proposed equal frequency resampling. However, further demand for km distance and/or sub-µm resolution will bring challenges to accurate frequency sweeping estimation. Increasing FUT length requires a longer optical path delay $L_{OPD}$ in the auxiliary interferometer. Although the Hilbert transform can overcome the Nyquist resample limitation, the accuracy of $\pi /2^{n}$ frequency interval in the Hilbert transform has to be guaranteed by high-speed DAQ. Moreover, the sub-µm spatial resolution needs a larger frequency sweeping range and higher tuning speed. All of these will eventually be decided by the capability of the hardware processing. The frequency in the auxiliary interferometer is $f_{AI}=\gamma nL_{OPD}/c$. With the 1000 m OPD length and 1000 nm/s sweeping speed, this frequency reaches up to 608 MHz. 

## 4. Conclusions

Extreme spatial resolution in optical frequency domain reflectometry not only provides precise position information along with the fibre under test, but it also enhances the signal-to-noise ratio of the backscattered light as well as the sensing accuracy (such as the Rayleigh backscattering spectrum shift). Inaccurate resampling during the estimation of nonlinear frequency sweeping distribution is the prominent noise for delivering theoretical-level spatial resolution. In this work, the proposed equal frequency resampling is able to suppress this noise and realise the ultimate spatial resolution. This will help to improve optical component metrology to μm-level detection and further enhance high-resolution sensing in the distributed optical fibre sensing area.

## Data Availability

The data presented in this study are available on request from the corresponding author. The data are not publicly available due to privacy.
